# Risk factors for lesions of the knee menisci among workers in South Korea’s national parks

**DOI:** 10.1186/s40557-016-0143-y

**Published:** 2016-10-10

**Authors:** Donghee Shin, Kanwoo Youn, Eunja Lee, Myeongjun Lee, Hweemin Chung, Deokweon Kim

**Affiliations:** 1 Department of Occupational and Environmental Medicine, Wonjin Green Hospital, 568-1 Sagajeong-ro 49-gil 53, Jungrang-gu, Seoul South Korea; 2 Department of Radiology, Wonjin Green Hospital, 568-1 Sagajeong-ro 49-gil 53, Jungrang-gu, Seoul South Korea; 3 Department of Orthopedics, Wonjin Green Hospital, 568-1 Sagajeong-ro 49-gil 53, Jungrang-gu, Seoul South Korea

**Keywords:** National park workers, Knee, Menisci lesions, Trekking, MRI

## Abstract

**Background:**

This study was designed to investigate the prevalence of the menisci lesions in national park workers and work factors affecting this prevalence.

**Methods:**

The study subjects were 698 workers who worked in 20 Korean national parks in 2014. An orthopedist visited each national park and performed physical examinations. Knee MRI was performed if the McMurray test or Apley test was positive and there was a complaint of pain in knee area. An orthopedist and a radiologist respectively read these images of the menisci using a grading system based on the MRI signals. To calculate the cumulative intensity of trekking of the workers, the mean trail distance, the difficulty of the trail, the tenure at each national parks, and the number of treks per month for each worker from the start of work until the present were investigated. Chi-square tests was performed to see if there were differences in the menisci lesions grade according to the variables. The variables used in the Chi-square test were evaluated using simple logistic regression analysis to get crude odds ratios, and adjusted odds ratios and 95 % confidence intervals were calculated using multivariate logistic regression analysis after establishing three different models according to the adjusted variables.

**Results:**

According to the MRI signal grades of menisci, 29 % were grade 0, 11.3 % were grade 1, 46.0 % were grade 2, and 13.7 % were grade 3. The differences in the MRI signal grades of menisci according to age and the intensity of trekking as calculated by the three different methods were statistically significant. Multiple logistic regression analysis was performed for three models. In model 1, there was no statistically significant factor affecting the menisci lesions. In model 2, among the factors affecting the menisci lesions, the OR of a high cumulative intensity of trekking was 4.08 (95 % CI 1.00–16.61), and in model 3, the OR of a high cumulative intensity of trekking was 5.84 (95 % CI 1.09–31.26).

**Conclusion:**

The factor that most affected the menisci lesions among the workers in Korean national park was a high cumulative intensity of trekking.

## Background

There are 21 national parks and 583 trails in South Korea, and annual visitors to these trails have increased from 40,958,773 in 2012 to 46,931,809 in 2013. To support visitors accounting for the entire population of South Korea, 1,105 national park workers patrol the trails and perform disaster prevention, safety management, visitor guidance, and rescue duties [1]. National park workers perform their work in nature itself owing to the work characteristics and are exposed to various safety and health hazards. Particularly, because they climb trails that are not level and their duties include weeding, pruning, installing or repairing equipment, and transporting rescued people, the incidence of work-related musculoskeletal disease may be high. However, previous reports are limited to the incidence of musculoskeletal symptoms of national park visitors and mountain climbers [2, 3], and there is a lack of research about the musculoskeletal diseases of national park workers who are more consistently exposed to danger than visitors or climbers. In one study about the association between musculoskeletal subjective symptoms and the type of work [4], 61.7 % of national park workers were symptomatic, a higher proportion than that of automobile industry workers (37.8 %) or aircraft maintenance mechanics (25.8 %) [5, 6]. Furthermore, considering that in a study of male professional climbers, 46 % had subjective musculoskeletal symptoms [7], the incidence of musculoskeletal diseases is high in national park workers, who climb more often than professional climbers. Considering the incidence of musculoskeletal symptoms according to body part, it is highest in legs/knees (38.3 %), followed by shoulders (32.8 %), waist (25.8 %), hands/wrists/fingers (18.0 %), arms/elbows (13.3 %), and neck (12.5 %).

National park workers are always exposed to the risk of musculoskeletal diseases owing to the nature of their work, and particularly, the incidence of musculoskeletal symptoms in the knee area is high. However, there is a lack of evaluation regarding the association between specific work factors and musculoskeletal diseases using objective tests rather than complaints of subjective symptoms. This study investigated the prevalence of menisci lesions in knees, which had a high rate of subjective musculoskeletal symptoms among body parts, in national park workers using magnetic resonance imaging (MRI). Moreover, demographic factors as well as work factors related to the menisci lesions were investigated through a survey. The menisci play a pivotal role in knee functions such as load sharing, pressure absorption, contact stress reduction, and safety maintenance [8], and evaluation of the menisci is important in knee diseases caused by chronic loads on the knees [9]. Therefore, menisci evaluation would be helpful in studying musculoskeletal disease of knees in national park workers.

This study was designed to investigate the prevalence of the menisci lesions in national park workers and work factors affecting this prevalence.

## Method

### Study population

The study subjects were 698 workers whose tenure was longer than 7 years among 1,105 workers who worked in 20 Korean national parks in 2014 (excluding Hallasan National Park managed by Jeju Special Autonomous Province). An orthopedist visited each national park and performed history taking and physical examinations. The subjects with knee symptoms and positive signs in McMurray test or Apley test were taken knee MRI scan. The knee MRI was performed in 124 national park workers.

All study subjects were informed of the purpose of this study. The subjects were consented to participate under the 'Ethics, consent & permissions' heading and to publish to report individual patient data. This study was approved by the Ethics Committee of Wonjin Institute for Occupational and Environmental Health. The reference number is IRB-2016-001.

### Study methods

#### Questionnaire survey

Demographic information such as sex, age, body mass index (BMI), smoking, alcohol drinking, regular exercise, and leisure activity as well as work-related factors such as tenure, overtime labor, holiday use, and the trekking times were investigated through a survey. Subjects were also asked whether they performed stair flight and kneeling movements within the past month and about previous knee injuries, previous knee pain, and past medical history.

#### High-signal grading of the menisci on knee MRI

Knee MRI was performed on 124 workers from November 2014 to February 2015. The MRI was taken to the workers with knee symptoms and positive physical examination. This study used 1.5 Tesla MRI (Philips MRI system, Achieva, Netherlands) and a knee joint coil, and measurement was conducted in the supine position with knee joint extension. The workers were examined using a standard six-sequence MRI protocol (axial, coronal, and sagittal T2-weighted Turbo Spin Echo (TSE) sequence; coronal proton density-weighted spectral presaturation with inversion recovery sequence; and coronal and sagittal proton density-weighted TSE sequence).

An orthopedist and a radiologist respectively read these images of the menisci of the knee joint using a grading system (grade 0–3) based on the MRI signals described by Crues et al. [10]: grade 0, no high signal in the menisci; grade 1, irregular type of high signal in the menisci; grade 2, linear type of high signal in the menisci; and grade 3, extension of high signal to the margin of the menisci. If the MRI scan area contained both knees, we used the result of the menisci with higher MRI signal grade as the dependent variable in the readings on the knees of the subjects.

Cohen’s kappa coefficient was calculated to evaluate consistency between readers. According to Viera AJ et al., a kappa coefficient of 0.61–0.80 means substantial agreement, indicating high consistency between two readers [11]. The kappa coefficient of the results read by two readers in this study was 0.622, corresponding to substantial agreement. If some knee MRI signal grade by the two readers did not match each other, the final judgment for the grade was determined under the agreement of them.

The grade 0 is no MRI high signal. The grade 1 is irregular MRI signal change in the menisci, not linear transformation [12]. In the case of grade 1, even if patients have symptoms on their knees, some surgery isn’t required. The diffuse degenerative process for this grade 1 is mainly observed in the arthroscopy as well. The study of Crues et al. classified the grade 1-2 in MRI signal of menisci as no torn, and the grade 3 was only as torn. Of course most radiologists interpret grade 3 as a tear of menisci, and more than 90 % of grade 3 signal require a procedure or surgery [13]. To mention of grade 2, however, the grade with knee symptoms can be observed the intrasubstance tear in the arthroscopy may require some surgery of menisci [14]. Moreover, in approximately 10 % of grade 2 signal, some tear of menisci can be seen on arthroscopy, which tends to be underestimated as it is usually on the posterior horn of the medial menisci [15, 16]. Accordingly, we judged that the classification between the grade 1 and grade 2 should be separated.

Finally, the grade 0 without MRI high signal and the grade 1 with irregular MRI signal change which may have diffuse degenerative process through arthroscopy were categorized to the same group. Meanwhile, the grade 2-3 with ‘the menisci tear’ in arthroscopy was classified as the other group, which may require surgery.

#### Classification according to location, site, and type of menisci tear

We investigated the classification of menisci tear according to location (medial and lateral), site (anterior horn, body, and posterior horn), type (longitudinal, horizontal, radial, and complex), and other factors (discoid menisci and root tear) of the tear among the subjects. If the location and the site of menisci tear were multiple regions, we only described and classified for the region that had more severe damage in the menisci. It may need detail descriptions for the menisci tear in order to find out the cause of occurrence of the tear.

#### Calculation of the cumulative intensity of trekking

To calculate the cumulative intensity of trekking of national park workers, the mean trail distance (km), the difficulty of the trail, the tenure at each national park (months), and the number of treks per month for each worker from the start of work until the present were investigated. The Korea National Park Service (KNPS) investigated the degree of the slope, width, distance, and road surface conditions for 1700 km of trails in national parks from 2011 to 2013 using Global Positioning System (GPS) measurements [17]. Based on this information, this study estimated the mean trail distance and the difficulty of the trails of the affiliated national parks. Also, information about the workplace and tenure (years) of workers was provided by the KNPS, and the mean trekking times per month in the corresponding parks were investigated through a self-administered survey.

**Fig. 1:**
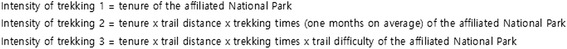
How to calculate the intensity of trekking

Based on the above data, the intensity of trekking was defined in three ways as shown in Fig. 1. The first method defined the intensity of trekking as the worker’s tenure at the affiliated national park, whereas the other two methods also took into consideration other factors such as distance and difficulty of trails. The intensity of trekking was calculated using the three methods for each national park, and the intensity of trekking of national parks workers from the beginning of their employment until 2014 was summed to calculate the cumulative intensity of trekking.

The cumulative intensity of trekking 1 was divided into two groups based on the tenure 17 years. 17 years was the average tenure of the workers in KNPS. Each cumulative intensity values of trekking 2 and 3 were divided into three parts by the two points on the basis of 33 % and 66 % of the distribution and they were classified into three groups: low, medium, high.

### Statistical analysis

First, we performed Chi-square tests to see if there were differences in the menisci lesions grade according to general characteristics (sex, age, BMI, smoking, alcohol drinking, regular exercise, previous knee injury, previous knee pain, and past medical history), occupational characteristics (overtime labor, holiday use, physical burden of work, and stair flight and kneeling movements), the classification of menisci tears, and the three ways of calculating the cumulative intensity of trekking.

Second, we evaluated the variables used in the Chi-square test using simple logistic regression analysis, and the crude odds ratio (OR) as well as 95 % confidence interval (CI) of the menisci lesions were calculated.

Finally, adjusted ORs and 95 % CIs were calculated using multivariate logistic regression analysis after establishing three different models according to the adjusted variables: model 1, adjusted for general and occupational characteristics and tenure; model 2, adjusted for general and occupational characteristics and the cumulative intensity of trekking 2; and model 3, adjusted for general and occupational characteristics and the cumulative intensity of trekking 3.

The significance level was set to <0.05.

## Results

### Characteristics of study population

The subjects of this study were mainly male, accounting for 93.5 %, and the mean age was 45.5 years. Less than half (39.5 %) of the workers had a BMI of ≥25 kg/m^2^; 48.4 % drank alcohol more than once a week, and 74.2 % were current smokers; 87.9 % regularly exercised more than once a week, and 37.9 % engaged in physical activities for leisure at least twice a week; 54 % had 7 to <17 years of tenure, and 46 % had ≥17 years. Moreover, 18.5 % of national park workers responded that they considered their workload was intolerable (Table 1).

**Table 1:** Characteristics of the subjects and the study populations by MRI signal grade of menisci (N = 124)

Variables		Number of subjects		MRI signal grade of menisci^a^		
				Grade 0-1	Grade 2-3	*P*-value^*^
		*N*	%	*N* (%)	*N* (%)	
General characteristics						
Sex	Male	116	93.5	48 (41.4)	68 (58.6)	0.361
	Female	8	6.5	2 (25.0)	6 (75.0)	
Age (year)	<45	66	53.2	32 (48.5)	34 (51.5)	0.048
	≥45	58	46.8	18 (31.0)	40 (69.0)	
Body mass index (BMI) (kg/m^2^)	<25	75	60.5	31 (41.3)	44 (58.7)	0.777
	≥25	49	39.5	19 (38.8)	30 (61.2)	
Smoking	Never or past	32	25.8	11 (34.4)	21 (65.6)	0.426
	Current	92	74.2	39 (42.4)	53 (57.6)	
Alcohol drinking	<1 times/week	64	51.6	23 (35.9)	41 (64.1)	0.304
	≥1 times/week	60	48.4	27 (45.0)	33 (55.0)	
Regular exercise	Once a week or more	109	87.9	46 (42.2)	63 (57.8)	0.250
	Less than once a week	15	12.1	4 (26.7)	11 (73.3)	
Leisure activity^b^	No	47	37.9	22 (46.8)	25 (53.2)	0.250
	Yes	77	62.1	28 (36.4)	49 (63.6)	
Previous exercise-related knee injury	No	90	72.6	37 (41.1)	53 (58.9)	0.771
	Yes	34	27.4	13 (38.2)	21 (61.8)	
Previous accident-related knee injury	No	78	62.9	36 (46.2)	42 (53.8)	0.085
	Yes	46	37.1	14 (30.4)	32 (69.6)	
Previous work-related knee injury	No	61	49.2	29 (47.5)	32 (52.5)	0.107
	Yes	63	50.8	21 (33.3)	42 (66.7)	
Previous knee pain	No	22	17.7	11 (50.0)	11 (50.0)	0.308
	Yes	102	82.3	39 (38.2)	63 (61.8)	
Past medical history^c^	No	86	69.4	39 (45.4)	47 (54.6)	0.086
	Yes	38	30.6	11 (28.9)	27 (71.1)	
Occupational characteristics						
Overtime labor	No	23	18.5	9 (39.1)	14 (60.9)	0.897
	Yes	101	81.5	41 (40.6)	60 (59.4)	
Holiday use	Yes	119	96.0	48 (40.3)	71 (59.7)	0.988
	No	5	4.0	2 (40.0)	3 (60.0)	
Physical burden of work^d^	Low	101	81.5	42 (41.6)	59 (58.4)	0.548
	High	23	18.5	8 (34.8)	15 (65.2)	
Stair flight^e^	No	92	74.2	41 (44.6)	51 (55.4)	0.103
	Yes	32	25.8	9 (28.1)	23 (71.9)	
Kneeling^f^	No	100	80.6	41 (41.0)	59 (59.0)	0.754
	Yes	24	19.4	9 (37.5)	15 (62.5)	
The cumulative intensity of trekking 1	<17 years	67	54.0	34 (50.8)	33 (49.2)	0.010
(= Tenure)	≥17 years	57	46.0	16 (28.1)	41 (71.9)	
The cumulative intensity of trekking 2^g^	Low	40	32.3	21 (52.5)	19 (47.5)	0.019
	Medium	55	44.3	22 (40.0)	33 (60.0)	
	High	29	23.4	7 (24.1)	22 (75.9)	
The cumulative intensity of trekking 3^h^	Low	42	33.9	23 (54.8)	19 (45.2)	0.006
	Medium	65	52.4	24 (36.9)	41 (63.1)	
	High	17	13.7	3 (17.6)	14 (82.4)	
MRI scan area	Left knee	59	47.6			
	Right knee	60	48.4			
	Both knees	5	4.0			
MRI signal grade of menisci^i^	0	36	29.0			
	1	14	11.3			
	2	57	46.0			
	3	17	13.7			
Location of menisci tear	No menisci tear	50	40.3			
	Lateral	22	17.8			
	Medial	52	41.9			
Site of menisci tear	No menisci tear	50	40.3			
	Anterior horn	11	8.9			
	Body of menisci	5	4.0			
	Posterior horn	58	46.8			
Type of menisci tear	No menisci tear	50	40.3			
	Longitudinal	0	0.0			
	Radial	29	23.4			
	Horizontal	43	34.7			
	Complex	2	1.6			

^a^Grading system based on the MRI signals decribed by Crues et al.

^b^Leisure activities requiring physical activity, Two or more times per week

^c^Rheumatoid Arthritis, Diabetes, Lupus disease, Gout, Osteoporosis, Menopause, Osteoarthritis, etc

^d^Low : tolerable workload, High : intolerable workload

^e^Last month an average of two more steps (36 cm) jump down the stairs at least 20 times for a day

^f^More than 2 h a day for 1 month average

^g^calculated with trail distance, employment period, trekking times

^h^calculated with trail distance, employment period, trekking times, trail difficulty

^i^All menisci were graded according to Crues JV II et al.: grade 0, no high signal in the menisci; grade 1, irregular type of high signal in the menisci; grade 2, linear type of high signal in the menisci; grade 3, extension of high signal to the margin of the menisci

^*^ *P*-value by chi-squared test

Within the past month, 25.8 % of workers climbed more than two stairs (height 36 cm) at least 20 times on average per day, and 19.4 % kneeled for at least 2 h. According to the cumulative intensity of trekking 2, 32.3 % were classified as low, 44.34 % as medium, and 23.4 % as high. According to the cumulative intensity of trekking 3, 33.9 % were classified as low, 52.4 % as medium, and 13.7 % as high.

In addition, 27.4 % of workers had previous knee injuries related to exercise, 37.1 % had traumatic knee injuries, and 50.8 % had work-related knee injuries. Furthermore, 82.3 % had previous knee pain, and 30.6 % of all workers had underlying.

### The relationship between characteristics and MRI high signal of menisci

We had taken the knee MRI to 124 subjects. 47.6 % of the MRI scan area were the left leg, 48.4 % were the right leg, and 4 % were the both legs.

According to the MRI signal grades of menisci, 29 % were grade 0, 11.3 % were grade 1, 46.0 % were grade 2, and 13.7 % were grade 3. Moreover, 59.7 % of workers undergoing knee MRI had grade 2 or higher menisci lesions. If the MRI scan area were both legs, we used the relatively higher grade result in this study.

We investigated the classification of menisci tear according to location, site, type, and other factors of the tear. 40.3 % in the total subjects were no menisci tear. For them with the menisci tear, 17.8 % were the lateral menisci tear and 41.9 % were the medial menisci tear. The posterior horn tear was the most common sites of menisci and the body tear was the lowest. Considering of the type of menisci tear, 23.4 % were the radial tear, 34.7 % were the horizontal tear, and 1.6 % were the complex tear. However, the workers with cyst, discoid menisci, menisci root tear, and longitudinal tear weren’t found in this study.

We investigated whether there were differences in the MRI signal grades of menisci according to demographic differences and differences in work characteristics. There was no difference according to sex. However, there was a statistically significant difference according to age, with the grade 2 or higher found in 69 % of workers aged 45 or older but in only 51.5 % of workers younger than 45 (*P* < 0.05). There were no differences in the MRI signal grades of menisci according to demographic characteristics such as BMI, smoking, alcohol drinking, regular exercise, and leisure activities. There was no significant difference with respect to overtime labor and holiday use. Regarding the physical burden of work, those who reported walking up stairs and kneeling within the past month had more lesions of grade 2 or higher, but the difference was not statistically significant. There were no differences in the MRI signal grades of menisci according to previous injuries. There was also no statistically significant in the analysis considering the factors such as the location, the site, and the type of menisci tear.

According to the intensity of trekking 1, which was based only on the tenure, the prevalence of grade 2 or higher menisci lesions was 71.9 % in those with ≥17 years tenure, which was greater than the 49.2 % prevalence in those with <17 years tenure. According to the intensity of trekking 2, for which the distance of trails as well as frequency of trekking in addition to tenure was considered, the prevalence of grade 2 and higher menisci lesions was 75.9 % in the high group, which was higher than that in the medium (60 %) and low (47.5 %) groups. According to the intensity of trekking 3, for which the difficulty of trails was additionally considered, the prevalence of grade 2 and higher menisci lesions was 82.4 % in the high group, which was higher than that in the medium (63.1 %) and low (45.2 %) groups. The differences in the MRI signal grades of menisci according to the intensity of trekking as calculated by the three different methods were statistically significant (*P* < 0.05) (Table 1).

Table 2 shows the crude ORs for the MRI signal grades of menisci. Regarding the demographic factor that affected the menisci lesions, the crude OR of age was 2.09 (95 % CI 1.00–4.37). Regarding the intensity of trekking, which affected the MRI signal grade of menisci, the crude OR of the cumulative intensity of trekking 1 was 2.64 (95 % CI 1.25–5.59), the crude OR of the cumulative intensity of trekking 2 was 3.47 (95 % CI 1.21–9.96) in the high group, and the crude OR of the cumulative intensity of trekking 3 was 5.65 (95 % CI 1.41–22.61) in the high group (Table 2).

**Table 2:** The relationship between characteristics and grade 2-3 in MRI high signal of menisci among workers in Korea national park service (*crude*)

Variables		*crude*
		OR^a^ (95 % CI^b^)
General characteristics		
Sex	Male	1
	Female	2.12 (0.41 ~ 10.94)
Age (year)	<45	1
	≥45	2.09 (1.00 ~ 4.37)
BMI	<25	1
	≥25	1.11 (0.53 ~ 2.32)
Smoking	Never or past	1
	Current	0.71 (0.31 ~ 1.65)
Alcohol drinking	<1 times/week	1
	≥1 times/week	0.69 (0.33 ~ 1.41)
Regular exercise	Once a week or more	1
	Less than once a week	2.01 (0.60 ~ 6.71)
Previous exercise-related knee injury	No	1
	Yes	1.13 (0.50 ~ 2.53)
Previous accident-related knee injury	No	1
	Yes	1.96 (0.91 ~ 4.23)
Previous work-related knee injury	No	1
	Yes	1.81 (0.88 ~ 3.75)
Previous knee pain	No	1
	Yes	1.62 (0.64 ~ 4.08)
Past medical history	No	1
	Yes	2.04 (0.90 ~ 4.62)
Occupational characteristics		
Overtime labor	No	1
	Yes	0.94 (0.37 ~ 2.38)
Holiday use	Yes	1
	No	1.01 (0.16 ~ 6.30)
Physical burden of work	Low	1
	High	1.34 (0.52 ~ 3.43)
Stair flight	No	1
	Yes	2.05 (0.86 ~ 4.92)
Kneeling	No	1
	Yes	1.16 (0.46 ~ 2.90)
The cumulative intensity of trekking 1	<17 years	1
(= Tenure)	≥17 years	2.64 (1.25 ~ 5.59)
The cumulative intensity of trekking 2	Low	1
	Medium	1.66 (0.73 ~ 3.77)
	High	3.47 (1.21 ~ 9.96)
The cumulative intensity of trekking 3	Low	1
	Medium	2.07 (0.94 ~ 4.55)
	High	5.65 (1.41 ~ 22.61)

^a^ *OR* Odds Ratio

^b^ *CI* Confidence Interval

To compare the effects of demographic factors with work-related factors on the occurrence of menisci lesions, multiple logistic regression analysis was performed for three models. In model 1, there was no statistically significant factor affecting the menisci lesions. In model 2, among the factors affecting the menisci lesions, the OR of a high cumulative intensity of trekking was 4.08 (95 % CI 1.00–16.61), and in model 3, the OR of a high cumulative intensity of trekking was 5.84 (95 % CI 1.09–31.26). Other factors did not show statistical significance (Table 3).

**Table 3:** The relationship between characteristics and grade 2-3 in MRI high signal of menisci among workers in Korea national park service (Adjusted)

Variables		Adjusted^a^		
		Model I^b^	Model II^c^	Model III^d^
		OR (95 % CI)	OR (95 % CI)	OR (95 % CI)
General characteristics				
Sex	Male	1	1	1
	Female	3.71 (0.48 ~ 28.66)	3.80 (0.48 ~ 30.41)	3.41 (0.42 ~ 27.58)
Age (year)	<45	1	1	1
	≥45	0.82 (0.19 ~ 3.63)	1.21 (0.46 ~ 3.17)	1.28 (0.51 ~ 3.21)
BMI	<25	1	1	1
	≥25	1.14 (0.49 ~ 2.67)	1.26 (0.53 ~ 2.95)	1.25 (0.53 ~ 2.94)
Smoking	Never or past	1	1	1
	Current	0.71 (0.26 ~ 1.98)	0.61 (0.21 ~ 1.74)	0.60 (0.21 ~ 1.72)
Alcohol drinking	<1 times/week	1	1	1
	≥1 times/week	0.99 (0.43 ~ 2.25)	0.85 (0.36 ~ 1.99)	0.90 (0.39 ~ 2.12)
Regular exercise	Once a week or more	1	1	1
	Less than once a week	2.78 (0.71 ~ 10.85)	2.76 (0.70 ~ 10.97)	2.73 (0.70 ~ 10.64)
Previous exercise-related knee injury	No	1	1	1
	Yes	1.00 (0.38 ~ 2.60)	1.09 (0.41 ~ 2.89)	1.03 (0.39 ~ 2.69)
Previous accident-related knee injury	No	1	1	1
	Yes	1.92 (0.78 ~ 4.70)	2.05 (0.83 ~ 5.06)	1.95 (0.79 ~ 4.82)
Previous work-related knee injury	No	1	1	1
	Yes	1.47 (0.64 ~ 3.38)	1.59 (0.68 ~ 3.70)	1.60 (0.69 ~ 3.74)
Previous knee pain	No	1	1	1
	Yes	1.21 (0.43 ~ 3.44)	1.21 (0.42 ~ 3.51)	1.22 (0.42 ~ 3.52)
Past medical history	No	1	1	1
	Yes	1.25 (0.45 ~ 3.46)	1.09 (0.39 ~ 3.06)	1.17 (0.42 ~ 3.24)
Occupational characteristics				
Overtime labor	No	1	1	1
	Yes	0.98 (0.34 ~ 2.83)	1.02 (0.35 ~ 2.94)	1.04 (0.36 ~ 3.03)
Holiday use	Yes	1	1	1
	No	0.63 (0.08 ~ 4.75)	0.56 (0.07 ~ 4.25)	0.65 (0.08 ~ 5.26)
Physical burden of work	Low	1	1	1
	High	0.80 (0.27 ~ 2.39)	0.67 (0.21 ~ 2.16)	0.66 (0.21 ~ 2.12)
Stair flight	No	1	1	1
	Yes	2.36 (0.79 ~ 7.04)	2.72 (0.90 ~ 8.26)	2.49 (0.83 ~ 7.53)
Kneeling	No	1	1	1
	Yes	0.82 (0.26 ~ 2.53)	0.62 (0.21 ~ 2.16)	0.59 (0.19 ~ 1.87)
The cumulative intensity of trekking	<17 years	1		
	≥17 years	2.81 (0.63 ~ 12.60)		
	Low		1	1
	Medium		1.79 (0.70 ~ 4.61)	2.02 (0.80 ~ 5.11)
	High		4.08 (1.00 ~ 16.61)	5.84 (1.09 ~ 31.26)

^a^Calculated by multiple logistic regression analysis

^b^Adjusted for general characteristics(sex, age, BMI, smoking, alcohol drinking, regular exercise, previous knee injury, previous knee pain, past medical history), occupational characteristics(overtime labor, holiday use, physical burden of work, stair flight, kneeling), the cumulative intensity of trekking 1 (= Tenure)

^c^Adjusted for general and occupational characteristics, the cumulative intensity of trekking 2

^d^Adjusted for general and occupational characteristics, the cumulative intensity of trekking 3

## Discussion

In this study investigating the menisci lesions of national park workers based on the grading system described by Crues et al., 59.7 % of the workers had the grade 2 or higher in MRI signal of the menisci. In a study of 85 people aged 40 years or more evaluating the menisci lesions according to the same grading system as our study, the incidence of grade 2 or higher lesions was 19.8 % in subjects <60 years of age and 40.6 % in those ≥60 years of age. In another study of 115 participants from a general population between 13 and 78 years old, the incidence of grade 2 or higher lesions was 28.2 %. Therefore, we found that the prevalence of the menisci lesions is higher in national park workers than in the general population, perhaps because their walking distance is greater than that of the general population owing to the nature of their work; furthermore, their work involves walking repeatedly uphill and downhill rather than on the level.

In this study, the cumulative intensity of trekking, which accounts for the greater workload, rather than demographic factors, in national park workers, statistically significantly increased the risk of the menisci lesions in multivariate regression analysis considering both demographic and work-related factors. However, it was difficult to find an article analyzing the effect of trekking of national park workers or professional climbers on the menisci lesions, but the burden of walking on the menisci could be estimated by evaluating the association between walking and the menisci lesions in nonprofessional long-distance runners. In a study of 26 nonprofessional long-distance runners who had at least 10 years of running experience with no previous history of knee trauma or knee surgery, 23.1 % had grade 2 or higher menisci lesions [18]. The reason the risk of menisci lesions was higher in national park workers was supposedly frequent walking on uneven ground and change of trekking pace owing to repeated uphill and downhill walking. Trekking long-distance uneven trails particularly stresses the knees of workers. Trekking more than 2 miles (3.2 km) increases the menisci lesions by 1.8 times [19], and particularly, downhill trekking can result in great loads on the lower limbs including knees and consequently increase the incidence of knee diseases [20].

Downhill trekking can cause loading on the knee joint, and in a previous study, downhill trekking of an 11° slope increased loading on the knee more than level walking [21]. Also, Andriacchi et al. compared the peak flexion moments of the lower limbs in various activities of daily life and reported that walking down a staircase caused four times more moment on the knee joint than other parts of the body [22]. Winter et al. reported that the peak knee joint moment increased by five times in downhill compared with level walking [23]. In the present study also, trail difficulty considering the slope and surface condition of the ground was analyzed in addition to other factors. In model 2, which did not take trail difficulty into consideration, a high cumulative intensity of trekking increased the risk of menisci lesions, but it increased even further by 5.84 times in model 3, which considered trail difficulty. This indicates that not only long-distance trekking but also the trail difficulty, which includes repeated uphill and downhill trekking, caused loading on the knees in national park workers and is a risk factor for the menisci lesions.

The study of Coggon D et al. showed that kneeling could increase the risk of knee disease. In their study for 518 patients who were listed for surgical treatment of knee, repetitive kneeling increased knee joint disease by 1.8 times [24]. Also, in a study for 625 Swedish who were engaged in works requiring frequent kneeling positions such as farming, forestry, construction industry, the risk of knee joint disease increased by 2.1 times compared with the general population of the same age group [25]. However, in the present study, kneeling did not statistically significantly increase the risk of menisci lesions. It was considered that the increase of knee loading due to specific positions such as kneeling was not great because national park workers do not often perform their work in this kind of position and trekking for facility management as well as trekking information service accounted for greater proportion of their workload.

In previous studies, demographic factors such as age and body weight in addition to work-related factors were shown to affect the menisci lesions. Anderson and Felson reported that the risk of knee osteoarthritis was increased 1.76-fold in males and 3.29-fold in females every decade [26]. Furthermore, Jerosch et al. reported that degeneration increased with age according to an evaluation of the degree of degeneration for menisci using the knee MRI signal grading system (grades 0–4) in 82 people who had no knee symptoms in the age range of 8–62 years [27]. In univariate analysis in the present study, the menisci lesions were statistically significantly increased in workers aged ≥45 years, but in multivariate analysis, it was not statistically significant. Moreover, age was not a significant factor to increase the menisci lesions when age was classified by decade (30s, 40s, and 50s or above). Unlike other studies, the effect of age on the menisci lesions of national park workers is relatively low because chronic injuries occur mainly in those ≥65 years of age in the general population and degenerative changes become greater as age increases [28], but the proportion of older workers is low as the mean age of national park workers is 45.5 years and most of them retired at the age of 57.

The American Medical Association(AMA) has reported that overweight or obesity is also a strong predictor of the menisci lesions [29]. In the study of Baker et al., in subjects aged 25–59 with tears of menisci found on arthroscopy, the OR was 2.3 (95 % CI 1.5–3.4) for a BMI of 24.1–27.0 kg/m^2^ and 1.7 (95 % CI 1.2–2.6) for a BMI of ≥27 kg/m^2^ [30]. In a study of subjects who had previous surgery of menisci, there was a statistically significant positive correlation between BMI and menisci lesions [31]. In the present study, there was no significant difference in the incidence of the menisci lesions according to BMI because the number of subjects with a BMI of ≥25 kg/m^2^ was small. Furthermore, we considered that obese workers did not participate as much in trekking owing to potential knee pain or attempted to control weight by themselves to reduce knee pain.

The menisci tears may be described by the factors such as location, site, and type of the tear. In this study, the medial menisci tear was the most part according to the location of menisci. In previous study as well, the medial menisci tear were more frequent than the lateral. Because the medial meniscus is ‘C’ shaped while the lateral is a shorter incomplete circle with closer spaced ‘horns’, but also since its more secure attachments to the tibia plateau make it susceptible to shear injury [32, 33].

Most menisci tear that occur in the young athletes is longitudinal. This tear is associated with ACL injury, and the medial menisci is more commonly affected for this tear [34]. However, in our study, the workers with longitudinal tears were not found. Radial tear is known to be more frequent in the lateral meniscus and can appear to be caused by athletic trauma [35]. While, in other study, radial tears occurring in the posterior horn of the medial meniscus were common [36]. The workers who had frequently trekked in our study could be damaged on the menisci by some trauma during the trekking, and the radial tear may occur to them. Horizontal tear is known as degenerative type and is more common in middle-aged people [37]. The posterior horn of the medial menisci is relatively no movable compared with other parts, and it’s weak to tear. Consequently, the horizontal tear of the posterior horn in the medial meniscus is the most emblematic of degenerative menisci tear [38]. They can occur in all age groups but increase with advancing years. On the other hand, the horizontal tear in the lateral menisci can occur to some runners [39]. Despite the average age of the subjects in our study was 44.3 years old and they retired from the KNPS in 55-59 years old, the ratio of horizontal menisci tear for them was the highest. We thought the menisci lesion by the long-period hikes similar to runners caused the tears.

This study has the following limitations. First of all, there was limitation to occur the bias in determining of the ambiguous MRI signal grade since the orthopedic surgeon directly carried out physical examinations to the subjects. Although the knee MRI was taken to the subjects with knee symptoms and positive signs in McMurray test or Apley test, the occurrence of bias in the readings of the reader who contacted to the workers directly was inevitable. In a further study, if the reader who does not contact to the subjects will read for the MRI signal grade, we think the study can increase the reliability of the readings.

Because this study was performed as a cross-sectional study, it was challenging to infer a causal association, and other results could be obtained given a different study period. Moreover, a prevalence-incidence bias could occur [40]. Additionally, because demographic and work-related factors were investigated using a self-administered survey, the intention of researchers and responders could be reflected. There was also a limitation of analytical results because 124 workers who underwent knee MRI were study subjects. A previous study evaluated not only the menisci lesions but also various diseases such as osteoarthritis of the knee, but our study did not evaluate factors related to knee diseases other than the menisci lesions. Another limitation could be that the statistically significant risk factor for the menisci lesions, the cumulative intensity of trekking, was estimated indirectly through trail distance as well as trail difficulty.

However, this was a unique study that was designed to objectively approach and evaluate the menisci lesions of workers in a specific environment, the national parks of Korea. Because various factors including demographic and work-related factors of national park workers were considered and analyzed, we could identify the strongest factor for increasing the risk of menisci lesions. Furthermore, in performing this study, reliability could be increased as an orthopedist visited workers individually, performed the physical examinations, and systematically classified the menisci lesions through the MRI high signals of the menisci. Regarding the evaluation of the cumulative intensity of trekking, not only trail distance but also trail difficulty such as slope angle and surface conditions were included and suggested that trail difficulty could affect the loading of the menisci.

Occupational musculoskeletal diseases occur owing to various factors in various types of work. Therefore, it is important to find and prevent risk factors at an early stage. The work-related factor that increased the menisci lesions in national park workers was the cumulative intensity of trekking in this study. Thus, to prevent the menisci lesions in national park workers, it is important to find a threshold of the cumulative intensity of trekking that causes injuries through a cohort study in the future. If workers who currently have no menisci lesions but a cumulative intensity of trekking that is greater than the threshold can be identified and advised to avoid trekking, the prevalence of menisci lesions of national park workers can be lowered.

## Conclusion

The factor that most affected the menisci lesions among the workers in Korean national park was a high cumulative intensity of trekking. It is necessary to establish a threshold of the cumulative intensity of trekking for knee disease and notify the workers who exceed this threshold and transfer them to other places with less trekking.

## Abbreviations

MRI: Magnetic resonance imaging
BMI: Body mass index
TSE: Turbo spin echo
KNPS: Korea National Park Service
GPS: Global positioning system
OR: Odds ratio
CI: Confidence interval
AMA: American Medical Association
